# Diagnosis, treatment, and monitoring of cytomegalovirus pneumonia in a hematopoietic stem cell transplantation child

**DOI:** 10.3389/fcimb.2025.1665477

**Published:** 2025-11-28

**Authors:** Fan Xuan, Chaonan Li, Hui Zhao, Na Liu, Xiaoqing Zhao, Baoxi Zhang, Xiaoli Wu

**Affiliations:** Department of Pediatrics, The Second Hospital of Hebei Medical University, Hebei, Shijiazhuang, China

**Keywords:** chronic myelogenous leukemia, hematopoietic stem cell transplantation, cytomegalovirus, bronchoalveolar lavage fluid, metagenomic next-generation sequencing

## Abstract

**Background:**

Cytomegalovirus (CMV), an opportunistic pathogen, can cause severe pneumonia in Chronic myeloid leukemia (CML) children undergoing hematopoietic stem cell transplantation (HSCT), resulting in a high mortality rate.

**Case presentation:**

An 11-year-old girl was hospitalized with a 3-day history of fever and vomiting, presenting with anemia and massive splenomegaly. A series of diagnostic tests, including blood cell count, bone marrow analysis, flow cytometry, chromosomal examination, and genetic testing, confirmed a diagnosis of CML at blast-phase. Following a one-year course of tyrosine kinase inhibitor-based chemotherapy, the patient entered the chronic phase and underwent a 6/12 human leukocyte antigen (HLA)-matched HSCT from her father. Two weeks after HSCT, the patient developed grade III skin graft-versus-host disease and hemorrhagic cystitis, which were effectively treated and symptoms were alleviated. One month after transplantation, the patient presented with serious pneumonia and pancytopenia. Although five blood cultures and two sputum cultures were all negative, metagenomic next-generation sequencing (mNGS) of bronchoalveolar lavage fluid (BALF) indicated a high abundance of CMV (16635 reads), leading to a diagnosis of CMV pneumonia. Notably, no typical resistant mutations were identified in the CMV genome. Targeted treatment with sodium phosphonoformate and letermovir was administered. As a result, the patient’s condition improved remarkably with the abundance of CMV decreasing to only 12 reads. After one-year of monitoring, the primary disease was well-controlled, and no CMV reactivation was observed.

**Conclusion:**

The diagnosis, treatment, and monitoring of pneumonia is crucial in post-HSCT patients. This case highlights the utility of mNGS in diagnosing and monitoring CMV pneumonia in post - HSCT patient and the effectiveness of targeted therapy in managing such infections.

## Introduction

Chronic myeloid leukemia (CML) is rare in the pediatric population, with a global incidence rate of 0.6-1.2 per million individuals. It accounts for 2-3% of all leukemias among children aged <15 years. CML with blast-phase presentation at diagnosis is more rare and aggressive in pediatric patients (Hijiya et al., 2016). Currently, allogeneic hematopoietic stem cell transplantation (HSCT) with prior chemotherapy remains the only curative therapeutic option for the majority of patients with CML in blast phase (Sembill et al., 2023). As the sole curative modality, HSCT is optimally performed in chronic phase but often complicated by CMV infection due to immunocompromise (Giménez et al., 2019; Senapati et al., 2023). HSCT recipients with refractory early post-transplant CMV infection have an elevated risk of CMV disease and non-relapse mortality (NRM) (Liu et al., 2015). Notably, CMV pneumonia, responsible for 40%-60% of post-transplant CMV diseases, is a critical driver of HSCT failure and recipient mortality, with a fatality rate of 30%-50% (Ljungman, 2008). Furthermore, prolonged and repeated use or subtherapeutic exposure of antiviral drugs may induce CMV resistance to standard therapies, elevating morbidity and mortality (El Chaer et al., 2016; Lynch and Märtson, 2025). Herein, we report a case of a pediatric patient with blast-phase CML at diagnosis, detailing the management of severe CMV pneumonia arising one month post-HSCT.

## Case report

**Figure 1:**
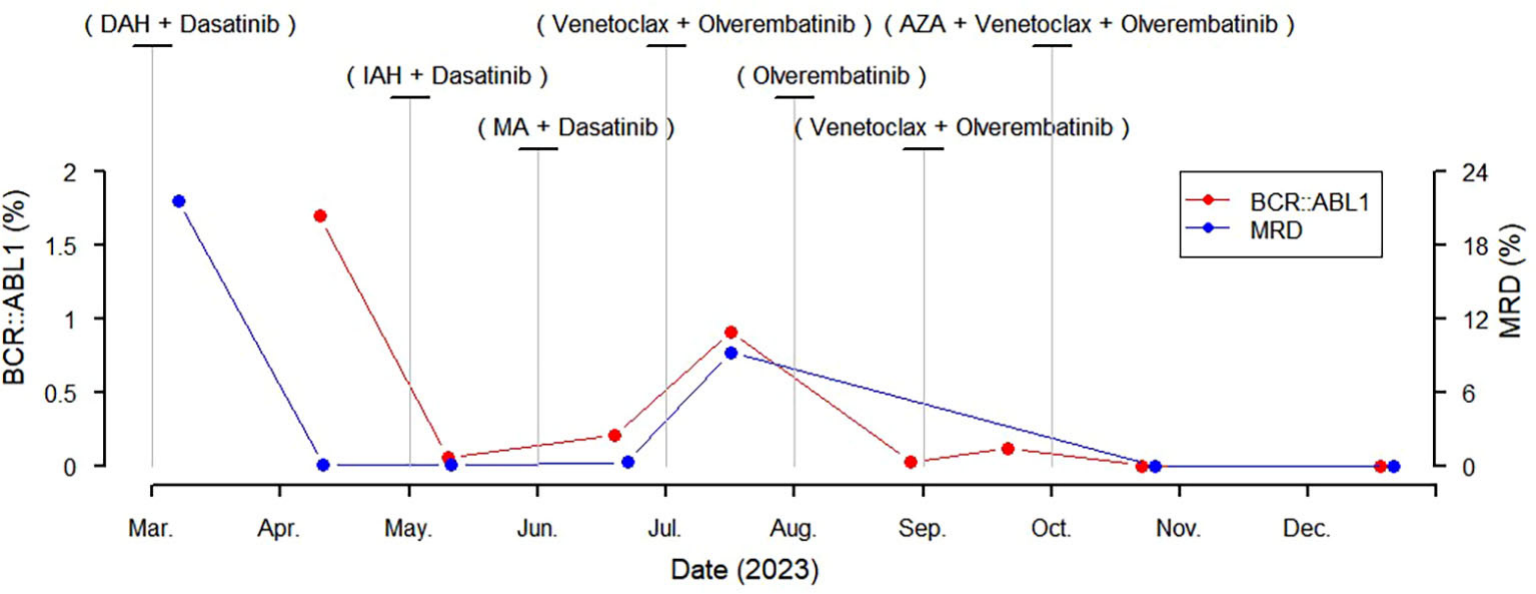
Treatment and monitoring of the patient with acute myeloid transformation of chronic myeloid leukemia. DAH, Daunorubicin + Cytarabine + Homoharringtonine; IAH, Idarubicin + Cytarabine + Homoharringtonine; MA, Mitoxantrone + Cytarabine; AZA, Azacitidine; MRD, minimal residual disease.

An 11-year-old girl was admitted to hospital due to fever and vomiting for 3 days. Physical examination revealed an anemic appearance and massive splenomegaly. Blood cell test showed a white blood cell count of 19.63e9/L, a hemoglobin level of 70 g/L, a platelet count of 223e9/L, and 31% immature cells. Blood biochemistry test showed a C-reactive protein level of 15.7 mg/L, a lactate dehydrogenase level of 1198 U/L, and a uric acid level of 423 μmol/L. No abnormalities were observed in coagulation function, autoantibodies, viral serology, thyroid function, complement, erythrocyte sedimentation rate, and immunoglobulins. Abdominal ultrasound indicated massive splenomegaly. Bone marrow cytology showed active proliferation of nucleated cells, increased blast cells accounting for 21.5%, and visible eosinophils. Bone marrow flow cytometry showed 21.6% abnormal myeloid blast cells expressing HLA-DR, CD13, CD33, CD34, and CD38, and some cells expressing CD117. Chromosome analysis revealed 46,XX,t(9;22)(q34;q11). Genetic testing showed positive BCR-ABL1 (P210), high expression of WT1, and high expression of EVI1. The patient was diagnosed with CML at blast phase. She was treated with tyrosine kinase inhibitor agents in combination with chemotherapy and entered the chronic phase after 1 year of treatment (**Figure 1**).

On April 3^rd^, 2024 (D0), the patient received a 6/12 human leukocyte antigen matched HSCT from her father. Laboratory tests before the transplantation showed that both the patient and her father were positive for CMV immunoglobulin G. The patient was administered methotrexate (MTX), cyclosporine, and mycophenolate mofetil for immunosuppression. On April 16^th^ (D + 13), the patient developed grade I acute graft-versus-host disease (aGVHD) involving the skin, presenting with pruritic rashes on the face and back. Methylprednisolone was initiated for anti-rejection therapy. On April 20^th^ (D + 17), the rashes exacerbated and spread to the neck, upper extremities, chest, back, buttocks, and bilateral thighs; immunosuppression therapy was continued. On May 3^rd^ (D + 30), the patient developed hemorrhagic cystitis. After hydration and diuretic treatment, clinical symptoms disappeared and laboratory test showed only microscopic hematuria. On May 5^th^ (D + 32), bullae appeared on both auricles, and the patient was diagnosed with grade IV cutaneous rejection. On May 10^th^ D(+37), the patient developed abdominal pain and hematochezia, with positive fecal occult blood on routine stool examination, leading to the diagnosis of grade IV intestinal rejection. Recombinant anti-CD25 humanized monoclonal antibody injection was administered for anti-rejection therapy on D(+34, +37, +41, and +48), while cyclosporine and methylprednisolone were continued. Abdominal pain and hematochezia resolved on May 20^th^ (D + 47). Methylprednisolone was tapered and discontinued on May 27^th^ (D + 54), and oral cyclosporine was maintained without new rash occurrence thereafter. During the immunosuppressive period, cyclosporine blood concentration was monitored regularly and adjusted to the appropriate range. During anti-CMV therapy, ruxolitinib was added for anti-rejection on June 8^th^ (D + 66) and tapered off on July 12^th^ (D + 100). Cyclosporine was tapered and discontinued on October 10^th^ (D + 190).

On May 17^th^ (D + 44), the CMV-DNA level in the peripheral blood was 1.47×10^4^ copies/mL. Sodium phosphonoformate was used for continuous antiviral treatment. Two days later, the patient developed cough and chest tightness. The percutaneous oxygen saturation was 92%, and no abnormalities was observed in the pulmonary imaging. Considering that the patient had previously suffered from fungal pneumonia, amphotericin B cholesterol sulfate complex was added for antifungal treatment. One week later, the copy number of CMV-DNA in the peripheral blood decreased to 3.24×10^3^ copies/mL, but the clinical symptoms was not alleviated. The patient presented with fever, cough, and chest tightness. With nasal cannula oxygen inhalation, the percutaneous oxygen saturation could be maintained at around 95%. As time went on, the cell count of platelet was decreased to 15×10^9^/L. Hypokalemia and hypoalbuminemia also occurred. With the decrease in the blood cells count (the cell count of platelet was decreased to 15×10^9^/L.), the patient’s hemorrhagic cystitis worsened significantly, and it turned into gross hematuria. Blood and sputum cultures were all negative, but the pulmonary CT scan showed diffuse interstitial lesions.

**Figure 2:**
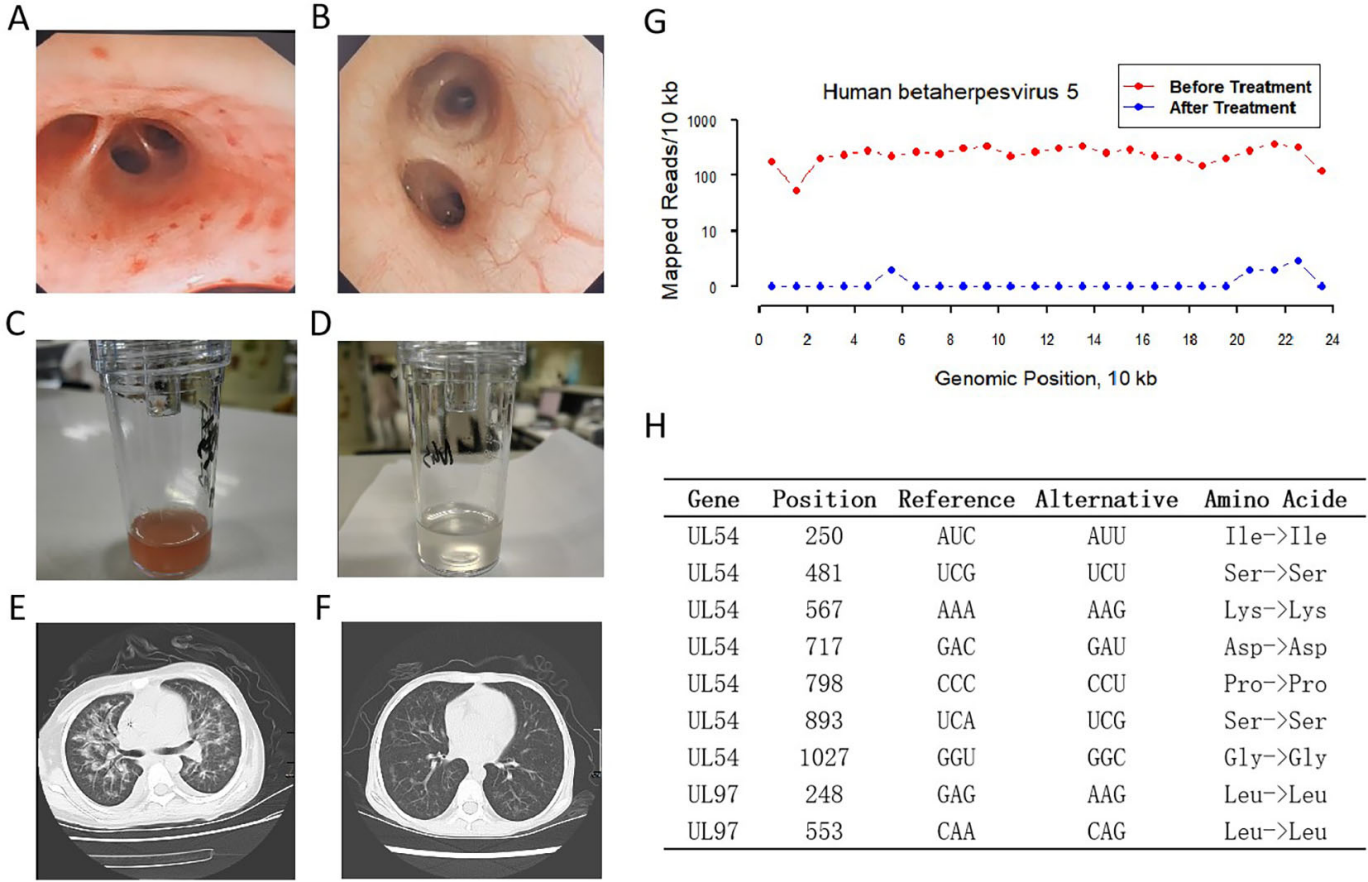
Clinical and laboratory findings for the management of CMV pneumonia in a HSCT child. Bronchoscopy images before **(A)** and after **(B)** treatment. BALF before **(C)** and after **(D)** treatment. Pulmonary CT before **(E)** and after **(F)** treatment. CMV coverage of the mNGS before and after treatment **(G)**. Mutations of UL54 and UL97 genes in the CMV genome **(H)**.

Bronchial lavage was performed for further diagnosis. Under the bronchoscope, multiple bleeding points were observed. The bloody lavage fluid was sent for metagenomic next-generation sequencing (mNGS), which revealed 16,635 sequences of CMV (relative abundance: 99.98%; average depth: 3.5394 ×) and 127 sequences of Candida albicans (relative abundance: 14.06%; average depth: 0.0004 ×). Additionally, enzyme-linked immunospot assay (ELISpot) demonstrated a low immune level of CMV-reactive T cells. The patient was diagnosed with CMV pneumonia with no typical resistant mutations identified in the sequenced CMV reads. The patient was then treated with sodium phosphonoformate in combination with letermovir for antiviral treatment. During the treatment, the patient’s temperature gradually returned to normal, and the oxygen saturation was well maintained without oxygen support. Two weeks later, the bronchoscopy showed that the airway mucosa was smooth and intact, and the lavage fluid was clear. mNGS showed that the sequence number of CMV was 12 (relative abundance: 1.35%; average depth: 0.0026 ×). During the treatment, the patient had a transient mild liver function impairment. The transaminase levels returned to normal after one week of liver protection treatment. There were no other complications. The number of blood cells gradually returned to normal after three weeks, and the patient was discharged from the hospital after the hemorrhagic cystitis was cured (**Table 1**). Three month later, no abnormalities were found in the pulmonary CT scan (**Figure 2**).

**Table 1:** Partial clinical and laboratory testing results of the patient in hospital (2024).

Clinical and laboratory testing	3-27 (D-7)	4-3 (D0)	4-17 (D + 14)	5-22 (D + 49)	6-1 (D + 59)	6-5 (D + 63)	6-12 (D + 70)	6-15 (D + 73)
Body temperature(°C)	36.4	37	37.2	38	37	36.6	36.3	36.6
White blood cell ([4.1-11.0×10^9^/L])	3.6	0.2	5.5	3.16	3.83	4.9	3.35	1.47
C-reactive protein ([0-10.0mg/L])	9.5	21.1	28.7	20.8	35	9.6	7.1	3.7
Red blood cell ([4.1-5.3×10^9^/L])	4.14	3.18	3.35	2.93	2.7	3.21	3.08	3.06
Hemoglobin ([114-154g/L])	114	88	93	95	85	101	97	96
Platelet ([150-407×10^9^/L])	73	83	38	38	15	27	32	29
Prothrombin ([9.4-12.5s])	12.8	12.1	26.5	22.6	12.7	13.6	12.3	12.5
D-Dimer ([0-0.243µg/ml])	0.6	0.03	0.06	0.15	0.34	0.26	0.23	0.15
Alanine Aminotransferase ([6-29U/L])	11.8	16.3	6.7	36	21.7	182.3	59.3	57
Aspartate Aminotransferase ([10-31U/L])	19	24.7	10.5	39	37.5	330	68.5	75
Creatinine ([33-75µmol/L])	37	19	25	25	41	40	31	38
K+ ([3.7-5.2mmol/L])	3.78	4.13	3.7	3.64	3.17	2.1	3.56	3.64
Procalcitonin ([0-0.25ng/ml])	0.05	0.65	0.55	0.05	0.09	0.06	–	0.06
Creatine kinase-MB ([0-5ng/ml])	15	17	18	10	17	16	23	10
Lactic dehydrogenase ([120-250U/L])	214	292	418	313	322	563	432	358
Myoglobin ([0-110ng/ml])	28	16	16	50.9	17.9	8.8	11.4	9.7
B-type natriuretic Peptide ([0-73pg/ml])	41	46	–	–	4245	2049	426	57.9
Blood culture	Neg.	–	Neg.	Neg.	Neg.	–	Neg.	–
Sputum culture	–	–	–	Neg.	Neg.	–	–	–

The patient underwent HSCT treatment on March 27^th^, 2024 (D-7) and received the hematopoietic stem cell infusion on April 3^rd^, 2024 (D0). Neg. referred to blood/sputum culture negative.

During the CMV infection period, the patient started oral letermovir on May 24^th^ (D + 51), and continued prophylactic administration of the drug until July 27^th^ (D + 115) after the resolution of CMV infection. Oral acyclovir was initiated on July 7^th^ (D + 95) for viral prophylaxis and has been continued to date. One year after HSCT, the patient’s primary disease was well controlled, the chimerism degree remained stable, and no CMV recurrent event occurred (**Table 2**). The last follow up was on July 29th, 2025 and the blood CMV-DNA test was negative.

**Table 2:** Clinical and laboratory testing results of out-of-hospital follow-up (2024 - 2025).

Clinical and laboratory testing	6-28 (D + 55)	7-5 (D + 93)	7-15 (D + 103)	8-12 (D + 131)	9-12 (D + 162)	10-12 (D + 192)	11-12 (D + 223)	2025-1-12 (D + 284)	2025-4-21 (D + 383)	2025-7-29 (D + 482)
Fever ([Yes/No])	No	No	No	No	No	No	N0	No	No	No
White blood cell ([4.1-11.0×10^9^/L])	3.42	3.72	1.78	7.2	3.7	4.3	4.02	1.99	2.32	2.76
C-reactive protein ([0-10.0mg/L])	0.9	8.59	5.4	3		10.46	2.2	3	3.0	3.0
Red blood cell ([4.1-5.3×10^9^/L])	3.24	3.22	2.77	4.2	4.11	3.58	2.69	3.55	4.47	4.51
Hemoglobin ([114-154g/L])	105	107	94	135	133	113	86	116	136	139
Platelet ([150-407×10^9^/L])	119	96	96	137	103	101	128	101	133	142
Alanine Aminotransferase ([6-29U/L])	36.7	39.9	68.8	33.9	37.7	92	188	50	31.3	64.6
Aspartate Aminotransferase ([10-31U/L])	51.9	49.6	61.7	62.9	45.8	54.2	30.7	42.6	31.6	59.8
Creatinine ([33-75µmol/L])	36	34	47	58.9	54	42.2	37	36	44	37
Creatine kinase-MB ([0-5ng/ml])	17	23	10	6	5	7	5	6	<0.18	0.39
Lactic dehydrogenase ([120-250U/L])	390	454	287	291.8	239.5	259.1	189	188	207	247
Myoglobin ([0-110ng/ml])	6.9	8.4	14.4	71.7	35.9	44.5	12.64	28.7	9.85	17.27
CMV-DNA ([<500copies/ml])	<500		<500			<500			<500	
MRD (%)			0			0		0	0	0
BCR-ABL1 (%)	0		0		0	0		0	0	0
Bone marrow chimerism rate (%)	99.74		99.79		99.83	99.79		99.92	99.65	99.83

## Discussion and conclusion

Pediatric CML with blast phase at initial presentation is both rare and clinical aggressive. The patient underwent one year’s treatment and monitoring before entering the chronic phase. When receiving HSCT treatment, the patient also developed skin graft-versus-host disease and hemorrhagic cystitis, which were appropriately treated and cured. The complexity and high risk of pediatric CML highlight the standard management according to the expert panel recommendations (How et al., 2021; Cross et al., 2023; Sembill et al., 2023).

CMV is the most common virus in post-HCST recipients. It belongs to the β-subfamily of the Herpesviridae family in humans and is the largest and most structurally complex virus within the Herpesviridae family. The vast majority of CMV infections are long-term latent infections. The virus can infect children through ways such as breastfeeding, blood transfusion, or vertical transmission. When the immune function is low or defective, CMV infection can lead to viremia and even be life-threatening (Cho et al., 2019; Prakash et al., 2021). The CMV immunoglobulin G was positive in the patient before HSCT but no CMV related diseases or symptoms were observed. However, the immune level of CMV-reactive T cells was low one month after the patient received HSCT.

Hemorrhagic cystitis is one of the common complications of HSCT, with the incidence of virus-associated hemorrhagic cystitis ranging from 10% to 30%. Common causative viruses include BK polyomavirus (BKV), adenovirus (ADV), human papillomavirus (HPV), John Cunningham virus (JCV), cytomegalovirus (CMV), human herpesvirus 6 (HHV-6), and simian virus 40 (SV40) (Cesaro et al., 2018; Gorczynska et al., 2005). The patient in this case developed hemorrhagic cystitis on D + 30, and CMV-DNA was detected in the peripheral blood on D + 44. Subsequently, the patient presented with gross hematuria during the CMV infection period. Hydration and diuretic treatment were administered, and the patient’s symptoms improved significantly by D + 81, though occult blood persisted. Before discharge, the patient’s CMV infection resolved and occult blood returned to normal. Therefore, the potential effect of CMV on hemorrhagic cystitis cannot be excluded.

In the diagnosis of CMV pneumonia, domestic and foreign guidelines still regard laboratory diagnoses, such as virus isolation, culture, histological microscopy, and polymerase chain reaction (PCR) titer detection, as the main criteria for diagnosing CMV pneumonia. The current diagnostic criteria in the United States are the standards formulated by the Infectious Diseases Society in 2016 (Ljungman et al., 2017), which are divided into two levels: proven CMV pneumonia and probable CMV pneumonia. Based on clinical manifestations and imaging features, etiological detection becomes the core of the diagnosis. The imaging manifestations of CMV pneumonia are diverse, and the changes of interstitial pneumonia such as centrilobular nodules and diffuse ground-glass opacities are the most prominent imaging features (Du et al., 2020). The gold standard for the diagnosis of CMV pneumonia is the detection of typical viral inclusions in lung biopsy (biopsy), or the detection of intracellular viruses through immunohistochemical staining and *in-situ* hybridization. However, lung biopsy has a relatively high risk, especially for those with poor platelet engraftment after hematopoietic stem cell transplantation. Moreover, the positive rate of virus culture is low and it takes a long time. In addition, patients with CMV pneumonia have an increased risk of secondary bacterial and fungal infections (Ueno et al., 2019). In this patient, given the negative blood and sputum culture results, it was difficult to consider CMV as the unique pathogen for the severe pneumonia.

With the continuous maturation and popularization of mNGS technology, it has provided a new and powerful method for the etiological diagnosis of patients with severe pneumonia (Zhang et al., 2025). For patients with impaired immune function, the probability of mixed infections with multiple pathogens in the lungs is significantly increased. Using mNGS technology to detect pathogenic microorganisms in the bronchoalveolar lavage fluid (BALF) of such patients can significantly improve the sensitivity and timeliness of diagnosis (Wang et al., 2019; Xu et al., 2023; Shen et al., 2023).

In immunocompromised patients, antibodies are often absent or delayed in appearance, and biopsy as well as pathogen isolation are technically challenging. Bronchoalveolar lavage offers advantages in diagnosing pulmonary complications following HSCT, while mNGS facilitates diagnosis in cases of negative cultures (Li et al., 2019). In the diagnosis and treatment of pediatric pneumonia, the combined diagnosis of mNGS and conventional methods reduced the mortality rate from 13.5% in previous studies to 2.68% (Yang et al., 2022). In this case, the child was in an immunodeficient state after hematopoietic stem cell transplantation and had a previous history of fungal pneumonia infection. Under the routine antibacterial and antifungal treatment, the clinical symptoms rapidly worsened, and there was a comprehensive decrease in the number of blood cells. With the aggravation of the infection, graft-versus-host disease (GVHD) might occur at any time. It was crucial for the prognosis of the child to quickly identify the pathogenic bacteria and strengthen targeted treatment (Abe et al., 2019). However, traditional etiological detection methods could not meet the clinical needs, and the mNGS detection of BALF pointed the way for subsequent treatment (Huang et al., 2021). The high number of CMV sequences with no resistant mutants confirmed the diagnosis of CMV pneumonia in the child. Ganciclovir monotherapy is the first-line treatment regimen for CMV pneumonia, and foscarnet sodium is an alternative regimen for patients intolerant to ganciclovir (Ljungman et al., 2019). In this study, due to the significantly decreased number of blood cells in the child, foscarnet sodium was administered for antiviral treatment. Previous research data showed that the adverse reactions of foscarnet sodium application were electrolyte disorders (hypokalemia, hypocalcemia) (Foolad et al., 2018). The child in this study developed hypokalemia, which was effectively relieved after active supplementation. Ganciclovir, valganciclovir, and foscarnet sodium are first-line therapeutic agents for the prevention and management of CMV infection post HSCT. In refractory CMV infection, expert consensus recommends combination therapy as second-line and third-line treatment (Cesaro et al., 2025). Letermovir is a CMV terminase complex inhibitor. It is effective and safe for prophylaxis of CMV infection in allogeneic HSCT recipients (Russo et al., 2024). In addition, letermovir is recommended as a second line therapy for post-transplant CMV infection in patients refractory to or resistant to ganciclovir/valganciclovir, enabling viral clearance or stabilization with a manageable safety profile (Phoompoung et al., 2020; Jorgenson et al., 2022; von Hoerschelmann et al., 2023). The child in this case developed severe CMV pneumonia, accompanied by chest tightness and decreased oxygen saturation. Thus, combination therapy with foscarnet sodium and letermovir was initiated to improve the patient’s clinical outcome. As a result, the child’s symptoms of pulmonary infection such as fever, cough, and low blood oxygen were quickly relieved, and the number of blood cells gradually increased. Finally, the imaging findings recovered. Currently, the child has not experienced CMV reactivation while taking acyclovir orally for antiviral treatment, and the underlying disease is well controlled.

In summary, given the high mortality rate and frequent co-infection in HSCT recipients, accurate diagnosis, treatment, and monitoring of CMV pneumonia in pediatric CML are of pivotal importance. mNGS facilitates early etiological identification, enabling clinicians to prescribe optimized anti-infective drugs, thereby improving patient outcomes.

## Data Availability

The datasets presented in this study can be found in online repositories. The names of the repository/repositories and accession number(s) can be found in the article/supplementary material.
